# Low serum uric acid levels and levodopa-induced dyskinesia in Parkinson's disease

**DOI:** 10.1055/s-0043-1761294

**Published:** 2023-03-14

**Authors:** Nayron Medeiros Soares, Gabriela Magalhães Pereira, Ana Carolina Leonardi Dutra, Nathalie Ribeiro Artigas, Júlia Schneider Krimberg, Bruno Elkfury Monticelli, Artur Francisco Schumacher-Schuh, Rosa Maria Martins de Almeida, Carlos Roberto de Mello Rieder

**Affiliations:** 1 Universidade Federal do Rio Grande do Sul, Faculdade de Medicina, Porto Alegre RS, Brazil.; 2 Hospital de Clínicas de Porto Alegre, Serviço de Neurologia, Porto Alegre RS, Brazil.; 3 Universidade Federal do Rio Grande do Sul, Instituto de Ciências Básicas da Saúde, Porto Alegre RS, Brazil.; 4 Universidade Federal do Rio Grande do Sul, Instituto de Psicologia, Porto Alegre RS, Brazil.; 5 Irmandade Santa Casa de Misericórdia de Porto Alegre, Serviço de Neurologia, Porto Alegre RS, Brazil.; 6 Universidade Federal de Ciências da Saúde de Porto Alegre, Departamento de Clínica Médica, Porto Alegre RS, Brazil.; 7 Universidade Federal de Ciências da Saúde de Porto Alegre, Curso de Física Médica, Porto Alegre RS, Brazil.

**Keywords:** Parkinson Disease, Uric Acid, Dyskinesias, Levodopa, Doença de Parkinson, Ácido Úrico, Discinesias, Levodopa

## Abstract

**Background** Levodopa is the most used and effective medication for motor symptoms of Parkinson disease (PD), its long-term use is associated with the appearance of levodopa-induced dyskinesia (LID). Uric acid (UA) is believed to play an important neuroprotective role in PD.

**Objective** To investigate if serum UA levels are related with the presence of LIDs in PD patients. Also, we investigated the associations among UA levels and clinical features of PD.

**Methods** We enrolled 81 PD patients (dyskinesia = 48; no dyskinesia = 33) in the present study. A blood sample was collected to evaluate serum UA levels, clinical evaluation included the following instruments: Montreal Cognitive Assessment (MoCA), Beck Depression Inventory II (BDI-II), MDS-Unified Parkinson's Disease Rating Scale (MDS-UPDRS), Hoehn and Yahr (HY), and the sub-item 4.1 of MDS-UPDRS IV (score ≥ 1). Additional relevant clinical information was obtained by a clinical questionnaire.

**Results** Serum UA levels were lower in the dyskinesia group when compared with the no dyskinesia group. The same result was found in the UA levels of both men and women. The multivariate analysis showed lower uric acid levels were significantly associated with having dyskinesia (odds ratio [OR] = 0.424; 95% confidence interval [CI]: 0.221–0.746; *p* = 0.005). Additional analysis verified that serum UA levels are inversely correlated with depressive symptoms, disease duration, MDS-UPDRS IV and time spent with dyskinesia. A positive correlation was found with age at onset of PD symptoms.

**Conclusions** The present study provides a possible role of serum UA levels in LID present in PD patients.

## Resumo

**Antecedentes** A levodopa é a medicação mais utilizada e eficaz para os sintomas motores da doença de Parkinson (DP); seu uso a longo prazo está associado ao aparecimento de discinesia induzida por levodopa (LID). Acredita-se que o ácido úrico desempenhe um importante papel neuroprotetor na DP.

**Objetivo** Investigar se os níveis séricos de AU estão relacionados com a presença de LID em pacientes com DP. Além disso, investigamos as associações entre os níveis de AU e as características clínicas da DP.

**Métodos** Foram incluídos 81 pacientes com DP (discinesia = 48; sem discinesia = 33) no presente estudo. Uma amostra de sangue foi coletada para avaliar os níveis séricos de AU, a avaliação clínica incluiu os seguintes instrumentos: Avaliação Cognitiva de Montreal (MoCA), Inventário de Depressão de Beck (BDI-II), MDS-Unified Parkinson's Disease Rating Scale (MDS-UPDRS), Hoehn and Yahr (HY) e o subitem 4.1 da MDS-UPDRS IV (escore ≥ 1). Informações clínicas relevantes adicionais foram obtidas por meio de um questionário clínico.

**Resultados** Os níveis séricos de AU foram menores no grupo com discinesia quando comparados ao grupo sem discinesia. O mesmo resultado foi encontrado nos níveis de AU de homens e mulheres. A análise multivariada mostrou que níveis mais baixos de ácido úrico foram significativamente associados a ter discinesia (odds ratio [OR] = 0,424; intervalo de confiança (IC) de 95%: 0,221–0,746; *p* = 0,005). Análises adicionais verificaram que os níveis séricos de AU estão inversamente correlacionados com sintomas depressivos, duração da doença, MDS-UPDRS IV e tempo gasto com discinesia. Uma correlação positiva foi encontrada com a idade de início dos sintomas da DP.

**Conclusões** O presente estudo fornece um possível papel dos níveis séricos de AU na LID presente em pacientes com DP.

## INTRODUCTION

Parkinson disease (PD) is a neurodegenerative disease characterized by the progressive death of dopaminergic neurons of the substantia nigra causing motor and nonmotor manifestations, which are susceptible to changes throughout the disease, depending on the stage of progression and pharmacological therapy. 1 Although the most used and most effective pharmacologic treatment for the motor symptoms of PD is levodopa, its effects are strong and paradoxical, as long-term use of this drug leads to the development of motor fluctuations and dyskinesia. 2 3

Levodopa-induced dyskinesia (LID) are involuntary hyperkinetic movement disorders and can be divided into three categories: (1) peak-dose dyskinesia (most common form, related to the moment the dose of levodopa in the blood peaks and reaches the best therapeutic effect); (2) diphasic dyskinesia (which occurs before or after the peak dose, regarding the rise and fall of levodopa in the blood); and (3) off-period dystonia (occurs in the period of the worst response to levodopa). 4 The appearance of these complications affects the management of the disease, besides being a source of disability to PD patients. 5

Although levodopa is necessary to the occurrence of LID, the severity of the disease determines its onset. A long-term clinical study has shown that the severity of PD has a fundamental role in the onset of LID even when the onset of the treatment with Levodopa happens early or late. 6 Thus, a proposal on the pathophysiology of LID involves the strong influence of dopaminergic presynaptic degeneration, that is, the denervation of the nigrostriatal pathway, associated with the first doses of levodopa, which, in turn, would sensitize the development of LID. 7

Oxidative stress is thought to play an important role in dopaminergic neurotoxicity. 8 Uric acid (UA), which is an important antioxidant present in blood and brain tissue, appears to have a neuroprotective effect on PD. 9 The PRECEPT study identified UA as an important molecular factor linked to neurodegenerative progress on PD, showing that patients, especially men, who have higher levels of serum UA, presented a lower percentage of loss of striatal [ ^123^ I]β-CIT, which is a marker for the presynaptic dopamine transporter, during the follow-up period. 10 This evidence suggests that UA might play a role on LID, since its emergence depends on the degeneration of pre-synaptic nigrostriatal cells and on the administration of levodopa. However, a recent study that investigated the role of UA on LID in PD patients found an oppositive result, showing that higher serum UA levels are associated with increased risk of LID in men with PD. 11

Given the evidence about the relation of nigrostriatal depletion with UA and, with the appearance of LIDs, we hypothesize that lower serum UA levels are related with the presence of LID in PD patients. Additionally, we explored for possible associations among serum UA levels and clinical features of PD.

## METHODS

### Participants

The present research was conducted in accordance with the Declaration of Helsinki and approved by the research ethics committee of the Hospital de Clínicas de Porto Alegre (HCPA, in the Portuguese acronym), Porto Alegre, state of Rio Grande do Sul, Brazil (protocol n° 2.106.230). The present study followed the Strengthening the Reporting of Observational Studies in Epidemiology (STROBE) guideline recommendations. 12

A total of 81 PD patients were recruited at the neurology ambulatory of the HCPA, located in Porto Alegre, RS, Brazil. Parkinson disease patients were diagnosed according to the London Brain Bank criteria. 13 Eligible participants met the following criteria: (i) not having severe cognitive decline; (ii) Hoehn and Yahr (HY) between I and IV points; (iii) do not have neurological comorbidities; (iv) do not have chemical or alcohol dependence and (v) do not have a deep brain stimulator.

All participants provided written consent after a full explanation of the procedures. After acceptance, the participants were submitted to a single individual interview with a trained researcher, who administered the scales and assessment questionnaires for each participant. A blood sample was collected to assess serum uric acid levels.

### Clinical assessments

A clinical and sociodemographic questionnaire was recorded to collect data about disease duration, age, sex, and body mass index (BMI). At the time of interview, a specialist evaluated PD participants during the ON-state of antiparkinsonian medication.

The cognitive screening and mood complaints were investigated through the Montreal Cognitive Assessment (MoCA) 14 and Beck Depression Inventory II (BDI-II), 15 respectively. To investigate the aspects related to PD, all patients were assessed using MDS-Unified Parkinson's Disease Rating Scale (MDS-UPDRS). 16 Severity of disease was measure with HY. Motor complications were assessed using MDS-UPDRS IV and dyskinesias by the sub-item 4.1 of MDS-UPDRS IV (score ≥ 1), which corresponds to the time spent with dyskinesia in PD patients.

Motor Phenotypes of PD were determined using MSD-UPDRS items to calculate mean tremor dominant (TD), postural instability/gait difficulty (PIGD), and indetermined scores. 17 The type of antiparkinsonian drug was described for each PD participant and the levodopa equivalent daily dose (LEDD) was calculated. 18

Serum Uric Acid was measured in blood samples collected during the interview with each participants interview using routine colorimetric testing. Blood samples were collected only between 12:00 pm and 17:00 pm. All analysis were conducted at Hospital de Clínicas de Porto Alegre.

### Statistical analyses

To assess the normality of data distribution, the Shapiro Wilk test was completed. The dyskinesia and no dyskinesia groups were compared using the unpaired t-test or the Mann-Whitney test, for quantitative variables, including UA levels, or the chi-squared test for categoric variables. These results are expressed as median, minimum, and maximum values with a 95% confidence interval (CI). Pearson or Spearman correlation were used in order to verify the relation among UA levels and the clinical features of PD. The association between UA levels and the risk of present dyskinesia in PD patients was assessed using a logistic regression model adjusted for disease duration, LEDD, and sex. For all analyses, statistical signiﬁcance was considered at *p* < 0.05. Statistical analyses were performed using IBM SPSS Statistics for Windows version 24 (IBM Corp., Armonk, NY, USA). The logistic regression graph was made using GraphPad Prism 8.0.1 (GraphPad Software, San Diego, CA, USA).

## RESULTS

**Figure 1:**
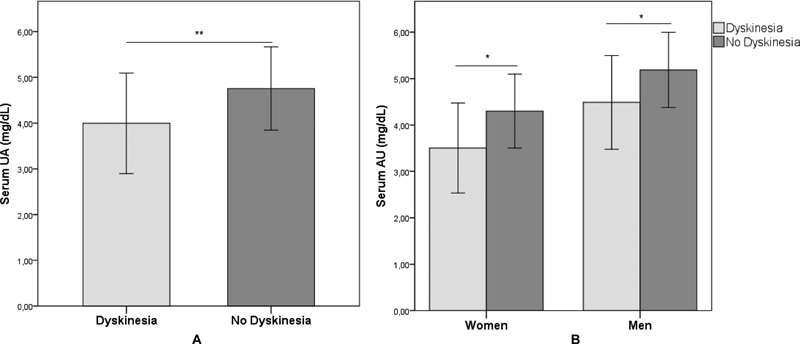
Serum uric acid levels in Parkinson disease (PD). ( **A** ) Overall dyskinesia group. ( **B** ) Dyskinesia group according to sex. Abbreviation: UA, uric acid.

Overall, PD patients had a mean age of 62.97 ± 9.83 years old and with similar distribution of men and women. A relation was found between levodopa-induced dyskinesias with a younger age, age at onset of PD, lower BMI, higher equivalent levodopa dose (LEDD), higher depressive symptoms, and higher total MDS-UPDRS score ( Table 1 ). The overall average of serum UA levels in PD patients and divided according to sex is described in Table 1 . Serum UA levels were different between groups, showing lower levels in the dyskinesia group when compared to no dyskinesia ( Table 1 ; Figure 1A ). The same result was found in the UA levels of both men and women ( Table 1 ; Figure 1B ).

**Table 1:** Clinical features of PD patients according dyskinesia

	Overall	Dyskinesia	No dyskinesia	p-value
**Men/Women**	41/40	24/24	17/16	0.537
**Age (years old)**	62.97 ± 9.83	59.44 ± 10.13	68.10 ± 6.71	< 0.001*
**Age at onset**	51.14 ± 10.50	47.00 ± 9.94	57.15 ± 8.23	< 0.001*
**Disease duration**	9.00 (1.00–29.00)	9.00 (2.00–29.00)	7.00 (1.00–28.00)	0.151
** BMI (kg/m ^2^ ) **	25.27 ± 4.13	24.07 ± 2.96	27.02 ± 4.96	0.004*
**LEDD**	1,050.00 (237–3,037.50)	1,106.25 (300.00–3,037.50)	800.00 (237.50–1,896.00)	0.008*
**MoCA**	25.00 (12.00–30.00)	25.00 (12.00–30.00)	25.00 (13.00–29.00)	0.383
**BDI-II**	16.00 (0.00–46.00)	19.00 (0.00–46.00)	13.00 (0.00–28.00)	0.045*
**MDS-UPDRS III**	44.84 ± 14.97	45.77 ± 15.11	43.48 ± 14.88	0.503
**MDS-UPDRS Total**	86.40 ± 29.53	94.52 ± 29.86	74.58 ± 25.05	0.002*
**HY**	2.00 (1.00–5.00)	2.00 (1.00–5.00)	2.00 (1.00–4.00)	0.003*
**TD**	30	11	19	0.003*
**PIGD and Indeterminate**	51	37	14	
**Serum UA (mg/dL)**	4.30 ± 1.08	3.99 ± 1.09	4.75 ± 0.90	0.001*
**Serum UA Men (mg/dL)**	4.77 ± 0.98	4.48 ± 1.01	5.18 ± 0.80	0.023*
**Serum UA Women (mg/dL)**	3.82 ± 0.97	3.50 ± 0.96	4.30 ± 0.79	0.010*

Abbreviations: BDI-II, Beck Depression Inventory II; BMI, body mass index; HY, Hoehn and Yahr stage; LEDD, Levodopa equivalent daily dose; MDS-UPDRS, Movement Disorder Society Unified Parkinson's Disease Rating Scale; MoCA, Montreal Cognitive Assessment; PIGD, postural instability/gait difficulty subtype; TD, tremor-dominant subtype. Data were expressed as absolute frequency, mean ± standard deviation, median and interquartile range.

Notes: Statistical analysis was performed as follows, Mann-Whitney test for BDI-II, Disease Duration, LEDD and MoCA; Unpaired student t-test for age, BMI, MDS-UPDRS and Uric Acid; Chi-square test for sex and motor subtypes (TD, PIGD and indeterminate).

No differences were found in sex, disease duration, MoCA and MDS-UPDRS-III according to the dyskinesia groups. There was a global association between having dyskinesias and motor subtypes [χ ^2^ (1) = 8.642; *p* = 0.003]. The analysis of adjusted residues showed that in the PIGD/indetermined subtype there was a greater proportion (3:2) in having dyskinesias when compared with the TD type ( Table 1 ).

Table 2 shows the correlation between serum UA levels and clinical features in PD. Serum UA levels were negatively correlated with depressive symptoms, disease duration, MDS-UPDRS IV and time spent with dyskinesia. A positive correlation was found between serum UA and age at onset of PD symptoms.

**Table 2:** Correlations among serum UA levels and clinical features of PD patients ( *n* = 81)

	r	*p-value*
**MoCA**	0.023 ^b^	0.837
**BDI-II**	−0.231 ^a^	0.046*
**Age at onset**	0,297 ^a^	0.007*
**Disease duration (y)**	−0.231 ^a^	0.038*
**LEDD**	−0.129 ^b^	0.252
**MDS-UPDRS III**	0.091 ^a^	0.418
**Freezing of gait (item 3.11)**	−0.151 ^b^	0.178
**MDS-UPDRS IV**	−0.226 ^b^	0.042*
**Time spent with dyskinesias (item 4.1)**	−0.296 ^b^	0.007*
**MDS-UPDRS Total**	−0.078 ^**a**^	0.487
**HY**	−0.153 ^**b**^	0.172

Abbreviations: BDI-II, Beck Depression Inventory II; MoCA, Montreal Cognitive Assessment; PD, Parkinson's Disease; HY, Hoehn and Yahr stage; LEDD, Levodopa equivalent daily dose; MDS-UPDRS, Movement Disorder Society Unified Parkinson's Disease Rating Scale.

Notes: Statistical analysis was performed using ^a^ Pearson's correlation for parametric distribution and ^b^ Spearman's correlation for non-parametric distribution.

**Figure 2:**
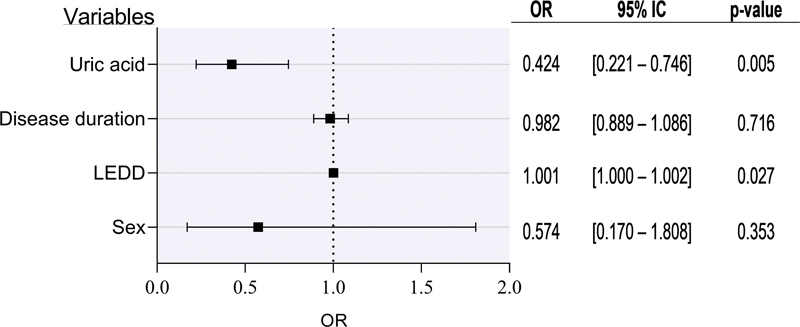
Logistic regression (OR) and 95% confidence interval (CI) of serum uric acid levels according to dyskinesia in Parkinson Disease (PD). For sex, women were the reference category. Abbreviation: LEDD, Levodopa Equivalent Daily Dose.

Figure 2 displays the results of a logistic regression that incorporated serum UA level, sex, disease duration and LEDD. After adjusting for these covariates, lower uric acid levels were significantly associated with having dyskinesia (odds ratio [OR] = 0.424; 95% confidence interval [CI]: 0.221–0.746; *p* = 0.005), which implies that, in this analysis, higher levels of UA are a protective factor for dyskinesia in this group. Sex, disease duration, and LEDD did not represent a significative result in this model.

## DISCUSSION

Generally, dyskinetic patients were characterized by having a young age at PD onset, low BMI, high LEDD, and increased disease duration, which is in line with previous studies. 19 20 21 People who develop PD at a younger age are at greater risk of developing LID, due to the combination of the duration of the disease and a higher cumulative dose of levodopa to control motor symptoms. 22 23 In addition, in dyskinetic PD patients, an increase in energy expenditure used in involuntary movements is common and seems to be the most important determining factor for weight loss, malnutrition, and lower BMI values in these patients. 24

PIGD motor subtype was the most common in this subgroup, in agreement with the argument that tremor symptoms predict lower development of LID in PD. 25 As main result, the present study demonstrated that there is a clinical relation between serum UA levels and LID in patients with PD. The PD dyskinesia general group, and the sex-specific PD dyskinesia subgroups had significantly lower serum UA levels when compared with the no dyskinesia groups. Furthermore, logistic regression confirmed that PD patients with lower levels of UA are more likely to present LID. These findings suggest that LID occurrence might be clinically associated with serum UA levels in both sexes, and PD patients with LID are more likely to present lower serum UA levels. Additionally, serum UA levels showed a negative association with other important clinical features; specifically: depressive symptoms, disease duration, motor complications, and time spent with dyskinesia in PD patients. A positive association between UA and the age of beginning of disease was found, indicating the possible relationship of UA in earlier development of PD.

Several clinical and epidemiologic studies have reported a neuroprotective effect of UA on PD progression. 10 26 27 28 Therefore, it is considered a potential biomarker of progression and risk of PD. 29 Our results appear to follow the same line, since low levels of UA presented a relation with having dyskinesia in PD. This makes sense, since LID depends directly on the levodopa peak-dose and degenerative progress of PD.

Pathophysiologically, the main hypothesis for LID is that its occurrence depends on a pulsatile stimulation of dopamine receptors (reached by levodopa administration), increased occupation of receptor due to large oscillation in presynaptic dopamine, dissociation between levels of intrastriatal and plasmatic dopamine, changes in postsynaptic neurons and abnormalities in other neurotransmitters, which lead to changes in firing patterns, disinhibiting thalamocortical neurons, and overactivating the motor cortex. 2 4 30 Considering this in association to the hypothesis that high UA levels might reduce the oxidative damage promoted by dopamine in the substantia nigra, 31 the mechanism of LID in PD patients might be affected by the low UA levels, worsening the oxidative damage on the nigrostriatal pathway.

The neuroprotective effect of UA in PD is predominantly observed in men, showing a sex dependence for the protective occurrence when compared with healthy individuals or in follow-up until the development of PD. 9 32 Physiologically, higher levels of UA occur in men when compared with women, ranging between 3.4–7.2 mg/dl and 2.4–6.1 mg/dl, respectively. 33 Apparently, it occurs in women due to a more effective renal clearance of UA promoted by estrogen during the premenopausal period, which might remain with the use of postmenopausal hormone. 34 35 In this sense, a previous study has not observed either relation of the risk of LID and UA levels in PD women. 11

Despite the literature, the results presented here suggest a relation between low UA levels and dyskinesia in PD women. In line with our results, an epidemiological cohort study suggested a slight inverse association between UA levels and the risk of DP in the female sex, but less significantly than in men 36 or only being observed after 8 years of follow-up. 37 A recent epidemiology study found an age-dependent neuroprotective effect of UA in women, showing this effect appears after 70 years old. 38 Furthermore, general data shows an increase in serum UA levels after 50 years or 70 years old, which is consistent with hormonal changes in the menopause period. 39 40 Based on these findings, our hypothesis is that once the neurodegenerative process is established and the critical premenopausal period is over, the UA might present a protective effect on PD women. Thus, in our dyskinetic sample, women were younger and, consequently, presented an earlier disease onset, suggesting a potential role of UA on LID in these women.

Additionally, it is important to report the inverse relationship among UA, depressive symptoms, and specific features of PD like disease duration and motor complications. Evidence have pointed to the worsening in several nonmotor and motor outcomes in PD patients with low UA levels. 10 41 42 These findings reinforce the potential role of UA in the severity of clinical symptoms and the progression of disease.

The present study has a few limitations. Although serum UA is a reliable biomarker, it may perform several biological functions and contribute to the pathophysiology of different diseases. Our sample consists of a single part of the population of PD patients in the south region of Brazil currently in treatment under the Brazilian unified public health system (SUS, in the Portuguese acronym). In our sample, the majority of the participants were Caucasian with average schooling and socioeconomic status, making it difficult to expand the results for the entire Brazilian population, since Brazil has a widely unequal socioeconomic distribution, which affects directly the nutritional status and access to antiparkinsonian medication. Also, the sample size was small and limits a generalization for all PD patients with LID. Although LEDD was collected from the medical record and confirmed by patients during the appointment, a small fraction of patients reported using different doses, which could influence LEDD in general. Patients might not have been aware of mild dyskinesias. However, the research team was prepared to clarify this symptom and look for any evidence of dyskinesias during the assessment.

In conclusion, the present study provides a possible role of serum UA levels in LID patients. Additionally, future longitudinal studies are encouraged in order to observe the continuous dynamics and to verify if low serum UA might be related with the onset of LID in PD.
